# Yap Regulates Müller Glia Reprogramming in Damaged Zebrafish Retinas

**DOI:** 10.3389/fcell.2021.667796

**Published:** 2021-09-20

**Authors:** Raquel Lourenço, Ana S. Brandão, Jorge Borbinha, Rita Gorgulho, António Jacinto

**Affiliations:** Chronic Diseases Research Centre (CEDOC), NOVA Medical School, NOVA University of Lisbon, Lisboa, Portugal

**Keywords:** zebrafish, retina, regeneration, Hippo-yap, Müller glia

## Abstract

Vertebrates such as zebrafish have the outstanding ability to fully regenerate their retina upon injury, while mammals, including humans, do not. In zebrafish, upon light-induced injury, photoreceptor regeneration is achieved through reprogramming of Müller glia cells, which proliferate and give rise to a self-renewing population of progenitors that migrate to the lesion site to differentiate into the new photoreceptors. The Hippo pathway effector YAP was recently implicated in the response to damage in the retina, but how this transcription coactivator is integrated into the signaling network regulating Müller glia reprogramming has not yet been explored. Here, we show that Yap is required in Müller glia to engage their response to a lesion by regulating their cell cycle reentry and progenitor cell formation, contributing to the differentiation of new photoreceptors. We propose that this regulation is accomplished through a *lin28a*–*ascl1a*-dependent mechanism, *bona fide* Müller glia-reprogramming factors. Overall, this study presents Yap as a key regulator of zebrafish Müller glia reprogramming and consequently retina regeneration upon injury.

## Introduction

Retinal degenerative diseases are frequently characterized by irreversible retinal degeneration leading to vision impairment or blindness. The search for novel therapeutic targets that stimulate endogenous repair mechanisms, restoring function in the context of disease and age-related biological processes, is thus of extreme importance. Interestingly, while mammals are not able to replace damaged or lost retinal neurons (Hamon et al., 2016), zebrafish have a remarkable ability to regenerate retinal cells after an insult (Lahne et al., 2020). Upon injury, zebrafish Müller glial cells (MGs) initiate a process of reactive gliosis, undergoing gene expression changes that prompt regeneration (Lahne et al., 2020). During this process, MGs are reprogrammed to adopt stem cell characteristics, reenter the cell cycle, and give rise to a pool of retinal progenitors that will differentiate into the missing cell types (Bernardos et al., 2007; Kassen et al., 2007; Nagashima et al., 2013). In contrast to zebrafish, mammalian MGs cannot reenter the cell cycle after retinal injury, precluding regeneration. Nevertheless, these MGs appear to have a dormant intrinsic neurogenic potential, being able to proliferate and generate other retinal cell types upon proper stimulation, which renders them as potential targets to be manipulated for regenerative therapies (Hamon et al., 2016).

A complex signaling network, which includes transcription factors such as Ascl1a, Lin28a, Stat3, and Sox2, has been proposed to be necessary for zebrafish MG reprogramming after retina injury. Knockdown of any of these factors results in reduced MG proliferation and impaired progenitor pool formation (Fausett et al., 2008; Ramachandran et al., 2010; Nelson et al., 2012; Gorsuch et al., 2017; Lust and Wittbrodt, 2018). However, how they work together to control MG reprogramming and retina regeneration is still not completely understood. Thus, to better understand how to potentiate retina repair, it is necessary to unravel how these transcription factors are regulated and how they cooperate in the molecular events that lead to an effective regenerative response.

The Hippo pathway kinase cascade and its nuclear effectors Yes-Associated-Protein 1 (YAP1) and WW-domain-containing transcription regulator 1 (WWTR1, also known as TAZ) have been associated with ocular disorders that often lead to blindness in mice and humans (Pocaterra et al., 2020). When the kinases are inactive, YAP/WWTR1 are not phosphorylated and can translocate into the nucleus to induce the transcription of genes that regulate diverse biological functions, including cell fate changes, proliferation, and tumorigenesis (Yu et al., 2015). The Hippo pathway has emerged as an attractive candidate to regulate organ regeneration across species, with YAP/WWTR1 activity being mostly associated with stem/progenitor cell expansion and inhibition of cell differentiation (Moya and Halder, 2019). Importantly, the Hippo pathway has recently been described to block mammalian MG reprogramming and cell cycle reentry by repressing YAP1, with YAP1 overactivation being sufficient to force MG proliferation (Hamon et al., 2019; Rueda et al., 2019). In addition, impairment of Yap1 activity by knockdown or through verteporfin treatment, in adult zebrafish, inhibits MG proliferation and photoreceptor regeneration (Hoang et al., 2020). Nevertheless, how Yap is integrated into the signaling network regulating MG reprogramming has not yet been described.

In this study, we further explore and characterize the requirement of Yap in regulating MG reprogramming using a genetic approach, namely, heat-shock (HS)-responsive transgenic lines that allow manipulating Yap function in a time-controlled manner (Mateus et al., 2015). We demonstrate that Yap activity inhibition impairs MG response to a light-induced lesion, leading to a reduction in MG proliferation, a lower number of progenitor cells, and impaired photoreceptor differentiation. Moreover, we propose a novel regulatory sequence of events where Yap regulates MG reprogramming by controlling the expression of *lin28a* and *ascl1a* transcription factors. Our results place the Hippo pathway effector as a central regulator of zebrafish retina regeneration after lesion and further supports the potential of Yap to be used as a therapeutic target to treat ocular disorders.

## Materials and Methods

### Zebrafish Lines and Maintenance

All zebrafish (*Danio rerio*) lines used were maintained in a recirculating system with a 10-h dark and 14-h light cycle at 28°C. Breeding of zebrafish strains was performed using standard procedures, and larvae were staged as described previously (Kimmel et al., 1995). Experiments were performed in several zebrafish transgenic lines: Tg(*hsp70*:RFP-DNyap) and Tg(*hsp70*:RFP-CAyap) (Mateus et al., 2015); Tg(*gfap*:GFP) (Bernardos and Raymond, 2006); and Tg(*careg*-EGFP) (Mateus et al., 2015; Pfefferli and Jaźwińska, 2017).

### Heat-Shock Experiments

For HS experiments, E3 medium was heated in a water bath at 37°C. To address the role of Yap during retina regeneration, dominant-negative and constitutively active Yap expression was induced by placing 6 days post-fertilization (dpf) DN-Yap, *careg*:eGFP;DN-Yap, *gfap*:eGFP;DN-Yap, CA-Yap, and *careg*:eGFP;CA-Yap larvae in the preheated medium and heat activation at 37°C for 30 min. Following the HS, larvae were returned to 28°C. A daily HS was induced, and larvae were left to develop until the desired stage.

### Total RNA Isolation and Quantitative Real-Time PCR

Total RNA was extracted from pools of 1 and 2 days post-lesion (dpl) larva eyes, from HS DN-Yap− controls and DN-Yap + embryos (50 larvae per condition), and four biological replicates obtained for each condition. RNA was extracted using TRIzol reagent (Invitrogen, Carlsbad, CA, United States) and the RNeasy Micro kit (Qiagen Inc., Valencia, CA, United States), according to the manufacturer’s protocol. cDNA was synthesized from 0.5 μg of total RNA with the Transcriptor High Fidelity cDNA Synthesis Kit (Roche Molecular Biochemicals, Mannheim, Germany) using a mixture of oligo dT and random primers. qRT-PCR was performed using a FastStart Essential DNA Green Master Mix and a Roche LightCycler 480. Cyclic conditions were as follows: 15 min preincubation at 95°C and three-step amplification cycles (50×), each cycle for 30 s at 95°C, 15 s at 65°C, and for 30 s at 72°C. For each biological replicate, three technical replicates were performed for each gene. Gene expression values were normalized using the *elongation factor 1*α (*ef1*α, NM_131263; *eef1a1l1*—Zebrafish Information Network) housekeeping gene, and fold change was calculated using the 2^–ΔΔ*C(T)*^ method (Livak and Schmittgen, 2001). Primer sequences are listed in Supplementary Table S1. Statistical analysis was performed using GraphPad Prism Software. Data groups were compared by paired *t*-tests. Only *p*-values < 0.05 were considered statistically significant.

### Retinal Light Lesions

Larvae with 6 dpf were exposed to high-intensity light to induce photoreceptor lesion as previously described (Meyers et al., 2012). Larvae were placed in a 50-ml glass beaker filled with 10 ml of E3 medium. The beaker was positioned 2 cm from the tip of a fiber optic light line connected to an EXFO X-Cite 120W metal halide lamp light source. Fish were exposed to intense UV light for 15 min, returned to a petri dish with embryo medium, and left to recover at 28°C.

### EdU Incorporation Assay

Ethynyl-2′-deoxyuridine (EdU; Thermo Fisher Scientific, Waltham, MA, United States; C10337) was added at 500 μM to embryonic medium [10 mM of stock solution in dimethyl sulfoxide (DMSO) diluted in 1× phosphate-buffered saline (PBS)], 6 h before larva fixation in 4% paraformaldehyde and processed for cryosectioning.

### Tissue Processing and Immunofluorescence

Larvae were fixed in 4% paraformaldehyde (PFA) overnight at 4°C, rinsed in PBS 1×, saturated in 20 and 30% sucrose (Sigma, St. Louis, MO, United States) in PBS 1× overnight and embedded in 7.5% gelatin (Sigma)/15% sucrose in PBS 1×, and mounted and subsequently frozen in liquid nitrogen. Longitudinal sections were obtained at 12 μm using a Microm cryostat (Cryostat Leica CM3050 S; Leica Biosystems, Wetzlar, Germany), and slides were maintained at −20°C.

Immunofluorescence on cryosections was adapted from Brandão et al. (2019) with the following modifications: after acetone permeabilization, slides were incubated in PBTD (PBS 1× with 0.1% Tween and 1% DMSO) for 15 min at room temperature (RT) with agitation and blocked for 2 h at RT in PBTD with 5% goat serum. After, they were incubated overnight at 4°C with primary antibody diluted in blocking solution (for antibody details, see Supplementary Table S2). When performing EdU staining, sections were incubated with the labeling solution according to the manufacturer’s protocol (Thermo Fisher Scientific: C10337). For the TUNEL labeling assay, sections were permeabilized in a sodium citrate solution (0.1% sodium citrate and 0.1% Triton X-100 in 1× PBS) and labeled according to the manufacturer’s protocol (Roche; 11684795910).

When using proliferating cell nuclear antigen (PCNA) antibody, an antigen retrieval step was required by incubating slides in heated sodium citrate buffer (10 mM of Tri-sodium citrate with 0.05% Tween 20, pH 6) solution for 20 min at 95°C. Coplin jars were then placed at RT, and slides were allowed to cool for 20 min. Slides were then rinsed in PBS 1× for 10 min and transferred to PBTD as described above.

### Image Analysis, Quantifications, and Statistical Analysis

Image acquisition of retina cryosections was performed in Zeiss LSM710 and LSM980 confocal microscopes at ×40 magnification using the software ZEN 2010B SP1. Retina 12-μm-thick z-stacks of 1,024 × 1,024 images were acquired with a step size of 0.5 μm. Images were processed using Fiji-ImageJ software (Schindelin et al., 2012), and maximum-intensity z-stack projections were generated. For all sections, the lens is on the left, and the dorsal is up.

Quantification of glutamine synthetase (GS) (in DN-Yap larvae), PCNA (in DN-Yap and CA-Yap larvae), green fluorescent protein (GFP) (in *careg*, DN-Yap larvae), and Sox2 (in *gfap*, DN-Yap larvae, and CA-Yap) positive cells was performed throughout the depth of the 12-μm retinal sections, in a minimum of three sections per eye, using the cell-counter Fiji plugin (for the number of retinas used on each assay, see Supplementary Table S3). The average number of positive cells was calculated per 100 μm^2^ of retinal area. The position of PCNA + and EdU + nuclei was determined in DAPI-labeled sections and cells grouped according to their position either in the inner nuclear layer (INL) or outer nuclear layer (ONL). The ciliary marginal zone (CMZ) was excluded from the count of proliferating cells. Statistical significance of the differences between control and manipulated larvae was assessed with unpaired *t*-tests with Welch’s correction using the Prism GraphPad software. Only *p*-values < 0.05 were considered statistically significant.

## Results

### Yap Inhibition Leads to an Accumulation of Activated Müller Glia Upon Photoreceptor-Induced Light Lesion

**FIGURE 1:**
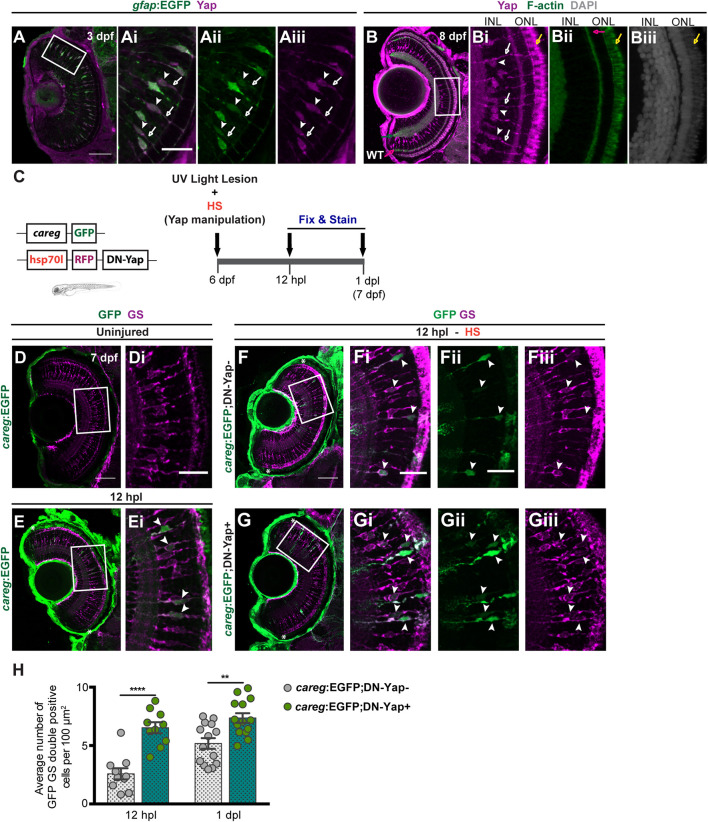
Yap inhibition induces accumulation of activated Müller glial cells (MGs) upon photoreceptor-induced light lesion. **(A)** Transverse cryosection of a Tg(*gfap*:GFP) 3-dpf retina immunostained for green fluorescent protein (GFP) (green, **Ai**) and Yap (magenta, **Aii**), indicating Yap localization in MG cell bodies (arrowheads) and radial processes (arrows) **(Ai–Aiii)**. **(B)** Transverse cryosection of a WT 8-dpf retina immunostained for Yap (magenta, **B,Bi**) and F-actin (green, **B,Bii**) and counterstained with DAPI (gray, **Biii**), indicating photoreceptors outer-segment autofluorescence (yellow arrow). **(C)** Schematic representation of the UV light lesion assay. **(D,E)** Transverse cryosections of uninjured 7-dpf *careg*-EGFP **(D,Di)** and 12-hpl *careg*-EGFP **(E,Ei)** larva retinas immunostained for GFP (green) and glutamine synthetase (GS) (magenta). White arrowheads indicate GFP-positive MGs activating *careg* (**E**, magnified, **Ei**). **(F,G)** Transverse cryosections of 12-hpl *careg*:EGFP;DN-Yap− controls **(F-Fiii)** and *careg*:EGFP;DN-Yap+ **(G-Giii)** retinas immunostained for GFP (green) and GS (magenta). White arrowheads indicate GFP-positive MGs activating *careg*. **(H)** Quantification of the number of double GFP GS-positive cells in *careg*:EGFP;DN-Yap− controls and *careg*:EGFP;DN-Yap + larva retinas from 12 hpl to 1 dpl. **p < 0.01, ****p < 0.0001; unpaired *t*-test with Welch’s correction. White boxes delimitate magnified **(Ai–Giii)**. Green arrow indicates the inner plexiform layer. Pink arrow indicates the outer plexiform layer. Scale bars correspond to 50 μm in **(A,D,F)** and 20 μm in magnified **(Ai,Di,Fi)**. Asterisks delimitate the lesioned region.

We started by characterizing Yap localization in the zebrafish retina at 3 dpf, using a previously validated Yap antibody (Brandão et al., 2019) and a glial fibrillary acidic protein (Gfap) reporter line (*gfap*:GFP) that labels MGs (Figure 1A; Bernardos and Raymond, 2006). Immunostaining indicates that Yap is localized in MG cell bodies and radial processes that extend apically and basally the INL (Figure 1A), from 3 dpf until at least 8 dpf (Figure 1B), suggesting that Yap may be required for normal retina development as previously reported (Asaoka et al., 2014; Miesfeld et al., 2015). Subsequently, we decided to address if it is necessary for retina regeneration. For that, we used a model of intense UV light lesion, described to damage only photoreceptors (Bernardos et al., 2007), and inhibited or overactivated Yap function in a time-controlled manner. This was done using HS-responsive transgenic lines expressing a dominant-negative (DN) and a constitutively active (CA) form of Yap, both fused to the RFP transgene and referred to as DN-Yap (hsp70l-RFP-DN-Yap) and CA-Yap (hsp70l-RFP-CA-Yap), respectively (Mateus et al., 2015). We subjected 6-dpf DN-Yap + and CA-Yap + larvae, with respective DN-Yap− and CA-Yap− controls, to an HS protocol, with both transgenes being detected in retina cells as early as 6 h post-HS (hpHS) (Supplementary Figures S1A–D). The HS does not compromise differentiated photoreceptor integrity (Supplementary Figures S1E–H) nor induces changes in retina cell proliferation when comparing DN-Yap + with DN-Yap− in an uninjured condition (Supplementary Figures S1K–N). To address the effects of Yap activity inhibition during regeneration, we subjected 6-dpf DN-Yap + and DN-Yap− larvae to a UV light lesion and to a daily HS protocol (Figure 1C). Upon light lesion, photoreceptors localized in the ONL gradually start to degenerate, as indicated by cell death labeling and scattered Zpr-1 (red/green-sensitive double-cone photoreceptor marker) staining at 1 dpl (Supplementary Figures S1I,J). Moreover, an increase in cell proliferation is also observed at 1 dpl, as shown by PCNA (G1/S cell cycle phase marker)-positive (PCNA+) cells within the retina, when compared with uninjured retinas (Supplementary Figures S1M–P).

Upon photoreceptor damage, MGs are activated and go through reactive gliosis (Lahne et al., 2020). To address the degree of MG activation upon lesion in zebrafish larvae, we used the *careg*:EGFP reporter (*ctgfa* reporter in regeneration). This reporter comprises a regeneration-specific regulatory element, shown to be upregulated in several cell types in response to injury (Mateus et al., 2015; Pfefferli and Jaźwińska, 2017). Considering this, whereas *careg* was not detected in uninjured larva retina (Figure 1D), *careg*-positive (*careg* +) MGs were observed as soon as 12 h post-lesion (hpl) (Figure 1E), as evidenced by colocalization of GFP with GS, an MG marker. This indicates that *careg* is also a suitable marker to detect the response and activation of MGs upon lesion in the zebrafish retina. To investigate whether Yap inhibition affects MG activation, we combined the DN-Yap with the *careg*:GFP (Figure 1C and Supplementary Figures S2A,B). Upon lesion, Yap inhibition led to an increase in the number of *careg* + MGs at 12 hpl and 1 dpl (Figures 1F–H), compared with DN-Yap− controls. On the contrary, Yap overexpression did not influence the number of *careg* + MGs at 12 hpl and 1 dpl (Supplementary Figures S2C–E), compared with CA-Yap− controls. These results suggest that in the context of Yap activity inhibition, MGs are able to perceive injury signals and that Yap may be necessary to regulate the activation status of MGs upon injury. This result goes in agreement with the study of Hoang et al. (2020), which states that *yap1* knockdown leads to an increase in reactive MGs in adult zebrafish.

### Yap Inhibition Reduces Müller Glia Injury-Dependent Proliferation

**FIGURE 2:**
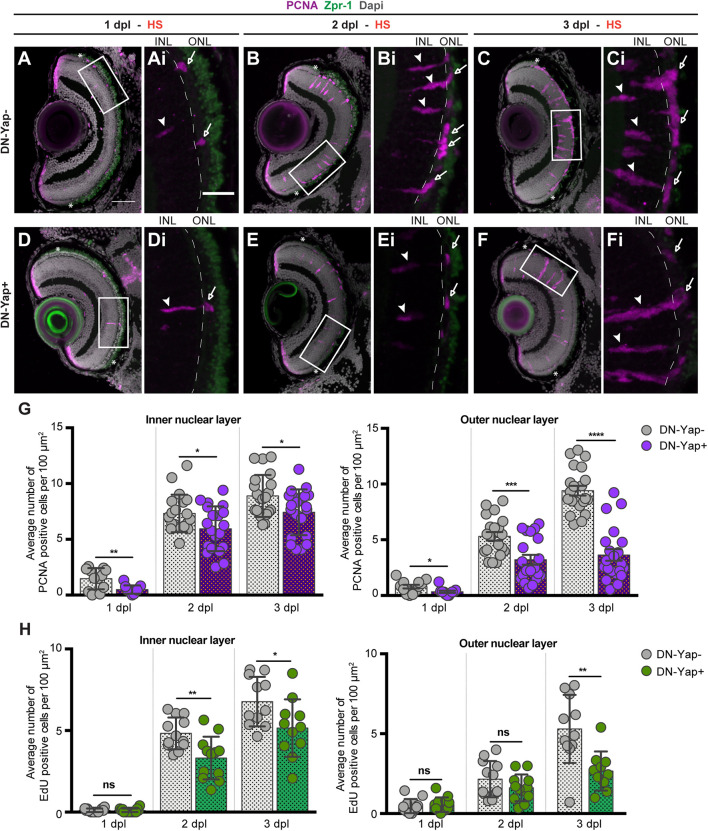
Yap inhibition reduces cell proliferation in the retina after photoreceptor-induced light lesion. **(A–F)** Transverse cryosections of DN-Yap− **(A–C)** and DN-Yap+ **(D–F)** retinas immunostained for proliferating cell nuclear antigen (PCNA) (magenta) and Zpr-1 (green) and counterstained with DAPI (gray). White arrowheads and arrows indicate PCNA+ cells in the inner nuclear layer (INL) and outer nuclear layer (ONL), respectively, at 1 dpl (**A,D** magnified, **Ai**,**Di**), 2 dpl (**B,E**, magnified, **Bi,Ei**), and 3 dpl (**C,F**, magnified, **Ci,Fi**). **(G)** Quantification of PCNA + cells in DN-Yap− and DN-Yap + larva retinas from 1 to 3 dpl, in the INL and ONL. **(H)** Quantification of EdU + cells in DN-Yap− and DN-Yap + larva retinas from 1 to 3 dpl, in the INL and ONL. ns, non-significant; *p < 0.05, **p < 0.01, ***p < 0.001, ****p < 0.0001. Unpaired *t*-test with Welch’s correction. Asterisks delimitate the lesioned region. Dashed lines delimitate INL from ONL. White boxes delimitate magnified **(Ai–Fi)**. Scale bars correspond to 50 μm in **(A,C)**, and 20 μm in magnified **(Ai,Ci)**.

Although MGs seem to be able to respond to the injury in Yap activity inhibition context, we proceeded to address whether Yap is required to correctly promote the MG regenerative response after light-induced damage. Upon lesion, MGs are reprogrammed to adopt stem cell characteristics, reentering the cell cycle and originating a pool of progenitor cells (Bernardos et al., 2007; Kassen et al., 2007; Nagashima et al., 2013). To investigate whether Yap is required for MG reprogramming, we evaluated the ability of MGs to reenter the cell cycle and proliferate by quantifying the number of PCNA + cells in lesioned retinas. From 1 to 3 dpl, PCNA + cells appear in the INL and ONL of DN-Yap− controls (Figures 2A–C) and DN-Yap + (Figures 2D–F) larval retinas. Proliferating cells correspond to MGs in the INL, as evidenced by colocalization of PCNA with GS, and MG-derived progenitors localized in the ONL (Supplementary Figures S3A,B). We observed a significant decrease in the number of proliferating cells in both INL and ONL regions of DN-Yap + retinas when compared with DN-Yap− controls (Figure 2G). We further confirmed this result using EdU (phase S marker) (Supplementary Figures S3C–E). A decrease in the number of proliferative EdU-positive (EdU +) cells (Figure 2H) was observed, indicating that fewer cells progress through the cell cycle in DN-Yap + retinas than in DN-Yap− controls. Additionally, we investigated whether Yap overactivation could induce the opposite phenotype. Supporting the results above, at 2 dpl, we observed more PCNA + MGs in the INL of CA-Yap + retinas, when compared with CA-Yap− (Supplementary Figure S4A). No differences were detected in the ONL, suggesting that cells stay arrested in the INL and are possibly unable to migrate to the ONL (Supplementary Figure S4A). These results indicate that Yap is necessary for MG cell cycle reentry and subsequent proliferation as well as for progenitor cell amplification.

The results are surprising considering that more MGs seem to be activated after blocking Yap transcriptional activity upon injury (Figure 1H). To explore the fate of the *careg*:EGFP + MGs in the context of Yap inhibition, we addressed the ability of activated MG (*careg* +) to proliferate in response to the light lesion. For that, we lesioned and HS *careg*:EGFP;DN-Yap larvae and perform immunostaining for PCNA at 2 dpl, when many cells are already proliferating (Figure 2D and Supplementary Figures S2G,H). In accordance with our previous results, the number of *careg* + MGs is still higher at 2 dpl in DN-Yap + retinas, compared with DN-Yap− controls (Supplementary Figure S2F). However, a reduction in the number of proliferating *careg* + cells is observed in the Yap activity inhibition context. This was demonstrated by quantifying the relative number of single *careg*:EGFP + and double *careg*:EGFP and PCNA + cells, in DN-Yap + retinas, when compared with DN-Yap− controls (Supplementary Figures S2G,H). These results indicate that *careg*:EGFP + MGs are less apt to proliferate, failing to properly contribute to the regenerative process. Thus, we hypothesize that injury-responsive MGs accumulate and become arrested in the Yap activity inhibition context, possibly as a means to compensate an unsuccessful proliferative response.

### Yap Inhibition Impairs the Formation of Müller Glia-Derived Progenitor Cells and Differentiation of New Photoreceptors After Lesion

**FIGURE 3:**
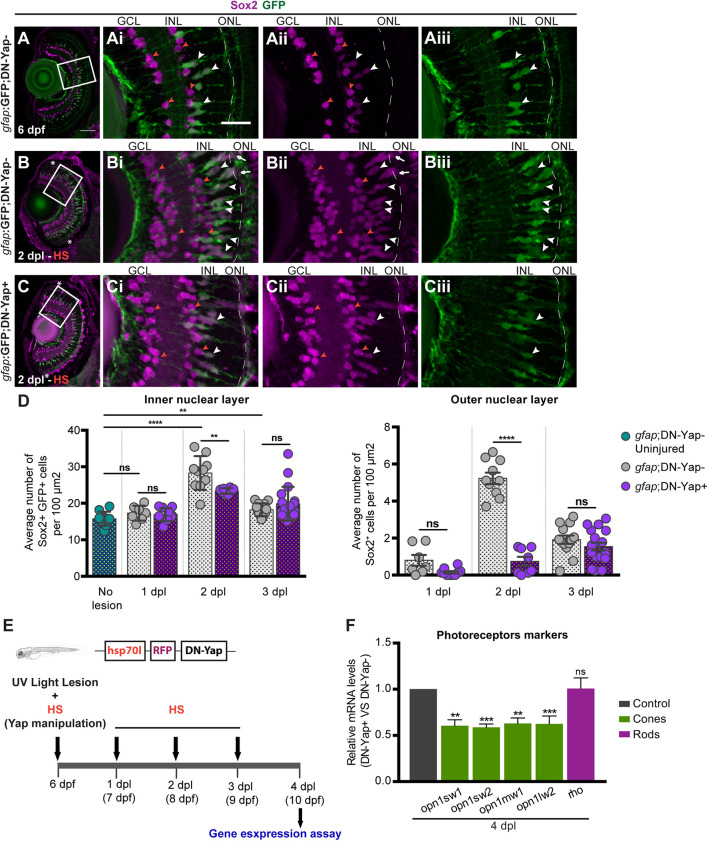
Yap is required to regulate the number of Sox2-positive Müller glia cell (MG) progenitor cells and expression of photoreceptor markers after photoreceptor-induced light lesion. **(A–C)** Transverse cryosections of uninjured 6-dpf *gfap*:GFP;DN-Yap− control retina **(A)**, 2-dpl *gfap*:GFP;DN-Yap− **(B)** and *gfap*:GFP;DN-Yap+ **(C)** retinas subjected to UV light lesion and heat shock (HS) at 6 dpf, immunostained for Sox2 (magenta) and green fluorescent protein (GFP) (green). Sox2 is localized in amacrine cells (ACs) (orange arrowheads) (magnified, **Ai,Aii,Bi,Bii,Ci,Cii**), MGs (white arrowheads), and progenitors in the outer nuclear layer (ONL) (white arrows) (magnified, **Ai–Aiii,Bi–Biii,Ci–Ciii**). **(D)** Quantification of Sox2 + GFP + cells in *gfap*:GFP;DN-Yap− uninjured controls at 6 dpf, and lesioned *gfap*:GFP;DN-Yap− and *gfap*:GFP;DN-Yap + larva retinas from 1 to 3 dpl, in inner nuclear layer (INL) and ONL. **(E)** Schematic representation of the UV light lesion and HS assay. **(F)** Relative gene expression of photoreceptor markers at 4 dpl (*n* = 7 biological replicates) in DN-Yap + versus DN-Yap− controls. ns, non-significant; **p < 0.01, ***p < 0.001, ****p < 0.0001. Unpaired *t*-test with Welch’s correction for Sox2+ cell quantification. Paired *t*-test for the relative gene expression of photoreceptor markers. Asterisks delimitate the lesioned region. Dashed lines delimitate INL from ONL. White boxes delimitate magnified **(Ai–Aiii,Bi–Biii,Ci–Ciii)**. Scale bars correspond to 50 μm in **(A)** and 20 μm in magnified **(Ai)**.

To further investigate the role of Yap after retina lesion, we evaluated the ability of MGs to generate new progenitor cells. Sox2, a neuronal stem cell-associated transcription factor, is expressed in MGs and amacrine cells (ACs) in the mature vertebrate retina (Taranova et al., 2006; Surzenko et al., 2013). Upon lesion, Sox2 expression is significantly increased in proliferating MGs, being required for MG proliferation and MG-derived progenitor amplification (Gorsuch et al., 2017; Lust and Wittbrodt, 2018). Therefore, we investigated whether Yap controls the emergence of the MG-derived Sox2-positive (Sox2+) progenitors after injury. For that, we subjected 6-dpf *gfap*:GFP;DN-Yap larvae, and respective *gfap*:GFP;DN-Yap− controls, to a light lesion. In uninjured controls, we observed Sox2 + MGs in the INL (Figure 3A). As expected, upon lesion, we detected more Sox2 + MG-derived progenitors in the INL and ONL in 2-dpl *gfap*:GFP;DN-Yap− retinas, when compared with the uninjured condition (Figures 3B,D). Strikingly, Yap inhibition leads to a major reduction in the number of Sox2 + MG-derived progenitors in both INL and ONL at 2 dpi, when compared with *gfap*:GFP;DN-Yap− controls (Figures 3C,D). At 3 dpl, differences are no longer observed, possibly reflecting a specific time window, at 2 dpl, when Sox2 + progenitor cells are required to repopulate the damaged areas and subsequently differentiate into new photoreceptors. Additionally, supporting the results above, Yap overactivation induced an increase in the pool of Sox2 + MG-derived progenitors in the INL of CA-Yap + retinas, when compared with CA-Yap− controls (Supplementary Figures S4B–D). In contrast, fewer Sox2 + MG-derived progenitors were detected in the ONL, supporting the idea that progenitors may be arrested in the INL and possibly unable to migrate to the ONL (Supplementary Figure S4D).

An important measure of the regenerative ability is the recovery of the cell types that were lost upon damage. To determine if inhibiting Yap function has a significant impact on photoreceptor regeneration upon light-induced lesion, we inhibited Yap function from 1 to 3 dpl and collected eyes at 4 dpl when regenerated photoreceptors start to differentiate (Figure 3E; Bernardos et al., 2007). We evaluated the expression levels of photoreceptor differentiation markers by assessing four classes of cone photoreceptors, based on the expression of different opsin proteins: *opn1sw1* (ultraviolet-sensitive), *opn1sw2* (blue-sensitive), *opn1mw1* (green-sensitive), and *opn1lw2* (red-sensitive) and rod photoreceptors, based on *rho* expression. At 4 dpl, we observed a significant reduction in the expression of all cone photoreceptor differentiation markers in DN-Yap + retinas relative to DN-Yap− controls (Figure 3F), indicative of less cone photoreceptor differentiation after damage. Conversely, rod photoreceptor differentiation appears to remain unchanged in the context of Yap inhibition (Figure 3F), suggesting that rod photoreceptor regeneration is being achieved by a resident population of late retinal progenitors known to give rise to rod photoreceptors in uninjured retinas (Bernardos et al., 2007). This suggests that Yap activity is necessary to specifically promote the recovery of cone photoreceptors after UV light-induced lesion. Overall, these findings support the hypothesis that, following retina injury, Yap is required for Sox2 + progenitor pool formation, amplification, and subsequent cone photoreceptor differentiation.

### Yap Inhibition Regulates the Expression of *lin28a* and *ascl1a* After Photoreceptor Light Lesion

**FIGURE 4:**
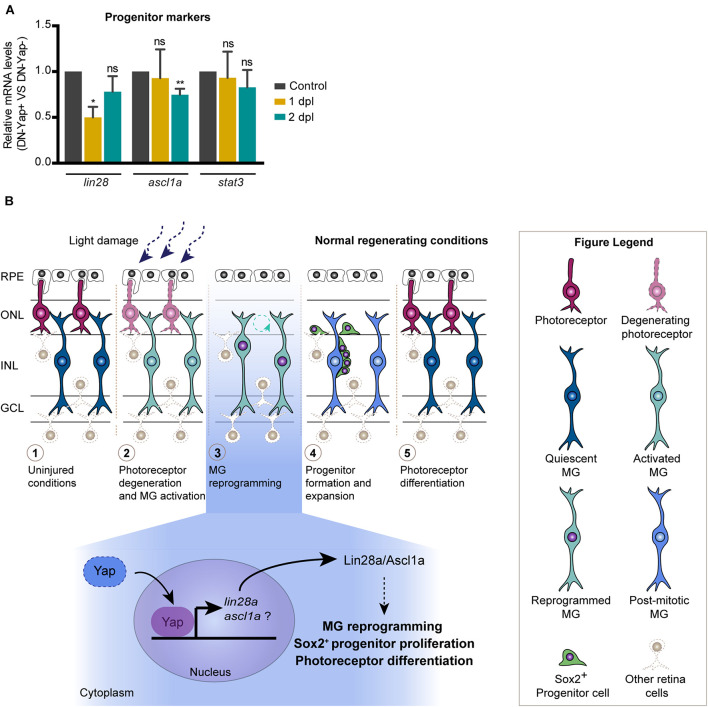
Model for the role of Yap during zebrafish retina regeneration. **(A)** Relative gene expression of progenitor markers at 1 and 2 dpl (*n* = 4 biological replicates for 1 dpl; *n* = 7 biological replicates for 2 dpl) in DN-Yap+ versus DN-Yap− controls. ns, non-significant; *p < 0.05, **p < 0.01. Paired *t*-test for the relative gene expression of progenitor markers. **(B)** In the zebrafish model system, in response to a light-induced lesion, photoreceptors start to degenerate, and quiescent Müller glial cells (MGs) become activated in response to the insult. Activated MGs then undergo a reprogramming event, reentering the cell cycle, and dividing and producing a pool of progenitor cells that migrate to the lesion site and differentiate into new photoreceptors. Based on our findings, we propose a model for the role of Yap during MG reprogramming after photoreceptor damage. Our data suggest that Yap is required to regulate MG reprogramming possibly via a *lin28a*–*ascl1a*-dependent mechanism, being necessary for correct Sox2 + progenitor proliferation and photoreceptor differentiation. We suggest that Yap regulates *lin28a* expression; however, if *ascl1a* is also regulated by Yap or Lin28a, it still needs further investigation.

Given that our data clearly point to an important role of Yap in inducing MG reprogramming and photoreceptor differentiation upon lesion, we set out to elucidate how Yap is integrated into the signaling events already described to participate in the regenerative response. For that, we addressed if Yap is able to control the expression levels of factors known to be necessary for MG reprogramming, namely, *lin28a*, *ascl1a*, and *stat3* (Fausett et al., 2008; Ramachandran et al., 2010; Nelson et al., 2012). We observed a significant reduction in *lin28a* expression at 1 dpl in DN-Yap + retinas relative to DN-Yap− (Figure 4A). Interestingly, *ascl1a* and *stat3* expression remained unaffected at this stage (Figure 4A). By 2 dpl, we observed a significant reduction in *ascl1a* expression, while *lin28a* and *stat3* expression was unaffected in DN-Yap + retinas relative to DN-Yap− controls (Figure 4A). These results show that Yap promotes *lin28a* expression at the onset of the injury response when MG cells undergo cell reprogramming, and *ascl1a* expression during the proliferative phase. In contrast, Yap does not appear to promote *stat3* expression, suggesting that its regulation is mediated through an alternative mechanism. Taken together, these results demonstrate that, after photoreceptor light lesion, Yap promotes MG reprogramming, and retina regeneration by interfering with the *lin28a*–*ascl1a* axis.

## Discussion

The Hippo pathway has been recently described to block mammalian MG reprogramming and cell cycle reentry by repressing its transcriptional coactivator YAP1, with YAP1 overactivation being sufficient to induce MG reprogramming into proliferative cells (Hamon et al., 2019; Rueda et al., 2019). Although these studies show a clear and relevant role of Yap in regulating proliferation in the neural retina, they are focused on the mouse-damaged retina, which does not possess a significant regenerative capacity. In a regenerative context, Yap has recently been shown to be required for MG lesion-dependent proliferation in *Xenopus* (Hamon et al., 2019) and photoreceptor regeneration in adult zebrafish (Hoang et al., 2020). In accordance with the later report, we show that Yap is also required to regulate MG cell cycle re-entry and proliferation during zebrafish larva retina regeneration. Importantly, in addition to what has been published, our work provides a more thorough analysis regarding the role of Yap in regulating crucial features of the zebrafish retina regenerative response. Our data indicate that Yap inhibition results in an accumulation of injury-responsive MGs, possibly to compensate for the inability of MG to reprogram and reenter the cell cycle, resulting in poor progenitor pool assembly and cone photoreceptor differentiation.

Despite the considerable amount of data on the relevance of Yap for retina regeneration, not much is known regarding the molecular mechanism by which YAP1 regulates the process. This has only started to be explored in the mouse damaged retina, with Hamon et al. (2019) work proposing the Yap−EGFR axis as a central player in MG response to injury. During zebrafish retina regeneration, Lin28a, a potent MG reprogramming factor, has been shown to induce Ascl1a expression to regulate MG proliferation (Nelson et al., 2012). Together with our data, which show that *lin28a* expression levels at 1 dpl and *ascl1a* at 2 dpl decrease in the context of Yap inhibition, this led us to propose that Yap might be a major regulator of retina regeneration by acting upstream of Lin28a, which in turn or in parallel with Yap regulates Ascl1a to induce the MG regenerative response, proliferation, and progenitor pool formation upon damage (Figure 4B). To support the idea that Yap is required for *lin28a* expression, evaluation of the *lin28a* promoter revealed a Tead1 binding motif (MatInspector software v3.13; data not shown), to which Yap typically binds (Yu et al., 2015). This is also consistent with recent findings indicating that Yap directly binds to this region of the *lin28a* promoter in the context of the zebrafish-injured lateral line and that a Yap−Lin28a axis reprograms sensory hair cell precursors to generate Sox2-positive progenitors (Ye et al., 2020). On the other hand, Sox2 has also been previously reported to promote MG reprogramming by controlling Lin28a and Ascl1 in the zebrafish regenerating retina (Gorsuch et al., 2017). Thus, we propose that Yap might play a role as an additional regulator of MG reprogramming and proliferation (Figure 4B). Importantly, additional work is necessary to further clarify if Yap and Sox2 might work together to promote MG reprogramming and retina regeneration after lesion.

In conclusion, we provide evidence that Yap is required to regulate the molecular events that promote zebrafish larval retina regeneration. We propose a model in which Yap is a regulator of MG initial response to the lesion. Particularly, our findings suggest that Yap emerges as an early key player in controlling zebrafish MG reprogramming upon lesion, possibly via a *lin28a*–*ascl1a*-dependent mechanism, which consequently leads to MG cell cycle reentry, progenitor cell formation, and photoreceptor differentiation. Our results point to an additional mechanism controlling retina regeneration so far not yet described. This work contributes to advance our knowledge on Yap integration in the signaling network regulating MG regenerative response, important for the development of strategies to unlock mammalian MG regenerative potential and counteract retina diseases.

## Data Availability Statement

The original contributions presented in the study are included in the article/Supplementary Material, further inquiries can be directed to the corresponding author/s.

## Ethics Statement

All the people involved in animal handling and experimentation were properly trained and accredited by FELASA. All experiments were approved by the ORBEA-NMS, Animal Use and Ethical Committees at Centro de Estudos de Doenças Crónicas (CEDOC), according to the European Union directives (Directive 2010/63/EU) and Portuguese law (Decreto-Lei 113/2013) for animal experimentation and welfare.

## Author Contributions

RL performed all the experiments with the help of AB, JB, and RG. RL and AB conceived and designed the experiments. RL performed data analysis. RL, AB, and AJ performed manuscript preparation. All authors critically reviewed the manuscript and approved the submitted version.

## Conflict of Interest

The authors declare that the research was conducted in the absence of any commercial or financial relationships that could be construed as a potential conflict of interest.

## Publisher’s Note

All claims expressed in this article are solely those of the authors and do not necessarily represent those of their affiliated organizations, or those of the publisher, the editors and the reviewers. Any product that may be evaluated in this article, or claim that may be made by its manufacturer, is not guaranteed or endorsed by the publisher.
