# Supplementary material for: Yap Regulates Müller Glia Reprogramming in Damaged Zebrafish Retinas
Source: Front Cell Dev Biol. 2021 Sep 20;9:667796. doi: 10.3389/fcell.2021.667796 (PMC8488126; doi:10.3389/fcell.2021.667796)
Supplement: Supplementary file 1 [file Data_Sheet_1.zip › Lourenco et al. 2021 Supplementary information.docx]

***Supplementary Material***

**Supplementary Figures**

**Supplementary Figure 1. Heat-shock assay does not affect differentiated photoreceptor or retina proliferation. (A-D)** Transverse cryosections of 6 dpf DN-Yap – **(A,Ai-Aiii)**, DN-Yap + **(B,Bi-Biii)**, CA-Yap – **(C,Ci-Ciii)** and CA-Yap + **(D,Di-Diii)** retinas subjected to a HS for 6 h, immunostained for RFP (magenta) and glutamine synthetase (GS) (green). DN-Yap – and CA-Yap – retinas show no RFP immunostaining (magnified panels Aii,Cii, respectively), while DN-Yap + and CA-Yap + retinas present transgenes expression, RFP-positive cells, through the different layers of the retina (magnified panels Bii,Dii, respectively). This is indicative of successful DN-Yap and CA-Yap transgenes induction upon HS and expression in MGs (white arrowheads in magnified panels Bi-Biii,Di-Diii, respectively). **(E-P)** Transverse cryosections of uninjured 6 dpf DN-Yap – controls **(E,Ei,K)** and DN-Yap + (F,Fi,L) retinas not subjected to HS; and uninjured 7dpf DN-Yap – control **(G,Gi,M)** and DN-Yap + **(H,Hi,N)** retinas subjected to a HS at 6 dpf. Retinas were immunostained for Zpr-1 (green) **(E-H)** or for proliferating cell nuclear antigen (PCNA) (magenta) **(K-N)**, and all counterstained with DAPI (grey). 1 dpl DN-Yap – control **(I,Ii,O)** and DN-Yap + **(J,Ji,P)** retinas subjected to UV light lesion and HS at 6 dpf. Retinas were labelled with TUNEL (magenta) and immunostained for Zpr-1 (green) **(I,J)** or immunostained for PCNA (magenta) **(O,P)**, and all counterstained with DAPI (grey). Pink asterisks indicate proliferating cells in the ciliary marginal zone (CMZ). White asterisks delimitate the lesioned region. White boxes delimitate magnified **(Ai-Hi,Ii-Pi)**. Scale bars correspond to 50 μm in **(A,E,K)** and 20 μm in magnified **(Ai,Ei)**.

**Supplementary Figure 2. DN-Yap and CA-Yap transgenes expression in activated MGs following HS. (A-D)** Transverse cryosections of 12 hpl *careg*:EGFP;DN-Yap – control **(A,Ai-Aiii)**, *careg*:EGFP;DN-Yap + **(B,Bi-Biii)**, *careg*:EGFP;CA-Yap – control **(C,Ci-Ciii)** and *careg*:EGFP;DN-Yap + **(D,Di-Diii)** retinas immunostained for RFP (magenta) and green fluorescent protein (GFP) (green). DN-Yap – and CA-Yap – retinas show *careg*:EGFP+ cells (orange arrowheads in magnified Ai,Aiii,Ci,Ciii respectively) with no RFP immunostaining (magnified Aii,Cii, respectively), while DN-Yap + and CA-Yap + retinas present transgenes expression, RFP-positive cells, colocalizing with *careg*:EGFP+ cells (white arrowheads in magnified Bi-Biii,Di-Diii, respectively). This is indicative of successful DN-Yap and CA-Yap transgenes induction upon HS and specific expression in activated MGs. **(E)** Quantification of the number of double GFP GS-positive cells in *careg*:EGFP;CA-Yap – controls and *careg*:EGFP;DN-Yap + larvae retinas from 12 hpl to 1 dpl. **(F)** Quantification of the number of double GFP GS-positive cells in *careg*:EGFP;DN-Yap – controls and *careg*:EGFP;DN-Yap + larvae retinas at 2 dpl. **(G)** Quantification of the number of GFP+PCNA- and GFP+PCNA+ cells in *careg*:EGFP;DN-Yap – controls and *careg*:EGFP;DN-Yap + larvae retinas at 2 dpl. **(H)** Percentage of GFP+PCNA- and GFP+PCNA+ cells in *careg*:EGFP;DN-Yap – controls and *careg*:EGFP;DN-Yap + larvae retinas at 2 dpl. ns, non-significant; **P < 0.01, ***P < 0.001, **** P < 0.0001; Unpaired *t-*test with Welch’s correction **(E,F)**. Mann-Whitney test in **(G,H)**. White boxes delimitate magnified **(Ai-Diii)**. Scale bars correspond to 50 μm in **(A)** and 20 μm in magnified **(Ai)**. Asterisks delimitate the lesioned region.

**Supplementary Figure 3. MGs proliferate after photoreceptor light lesion and upon Yap inhibition. (A-B)** Transverse cryosections of 2 dpl DN-Yap – control **(A,Ai-Aiii)** and DN-Yap + **(B,Bi-Biii)** retinas immunostained for proliferating cell nuclear antigen (PCNA) (magenta) and glutamine synthetase (GS) (green). Proliferating MGs are observed in the inner nuclear layer (INL) (white arrowheads) and in the outer nuclear layer (ONL) near MG endfeet (arrows) **(magnified Ai-Biii)**. **(C)** Schematic representation of EdU administration to the medium in the UV light lesion assay, 6 h before larvae fixation. **(D,E)** Transverse cryosections of 2 dpl DN-Yap – controls **(D,Di-Diii)** and DN-Yap + **(E,Ei-Eiii)** retinas immunostained for EdU (green) and PCNA (magenta). EdU and PCNA-double positive proliferating cell are observed in the INL (white arrowheads) and in the ONL (arrows) **(magnified Di-Eiii)**. Asterisks delimitate the lesioned region. Dashed lines delimitate INL from ONL. White boxes delimitate magnified **(Ai-Biii; Di-Eiii)**. Scale bars correspond to 50 μm in **(A,D)** and 20 μm in magnified **(Ai,Di)**.

**Supplementary Figure 4. Yap overexpression increases the number of proliferating MGs and Sox2-positive cells after photoreceptor light lesion. (A)** Quantification of proliferating cell nuclear antigen (PCNA)-positive cells in lesioned CA-Yap – controls and CA-Yap + larvae retinas at 2 dpl, in both inner nuclear layer (INL) and outer nuclear layer (ONL). **(B-C)** Transverse cryosections of 2 dpl CA-Yap – control **(B)** and CA-Yap + **(C)** retinas subjected to UV light lesion and HS at 6 dpf, immunostained for Sox2 (magenta) and glutamine synthetase (GS) (green). Sox2 is localized in ACs, in both GCL and INL (orange arrowheads) **(magnified Bi,Bii,Ci,Cii)**, MGs in the INL (white arrowheads) and in progenitors in the ONL (white arrows) **(magnified Bi-Biii,Ci-Ciii)**. **(D)** Quantification of Sox2-positive cells in lesioned CA-Yap – controls and CA-Yap + larvae retinas from 1 to 3 dpl, in both INL and ONL. ns, non-significant; *P  0.05, **P  0.01, *** P  0.001; Unpaired *t*-test with Welch’s correction. White boxes delimitate magnified **(Ai-Aiii,Bi-Biii)**. Scale bars correspond to 50 μm in **(A)** and 20 μm in magnified **(Ai)**. Asterisks delimitate the lesioned region.

**Supplementary Tables**

**Supplementary Table 1. Sequences of primers used for qRT-PCR analysis**

| **Gene**  **Ref Ensembl (*Danio rerio*)** | **Forward (5’>3’)** | **Reverse (5’>3’)** |
| --- | --- | --- |
| ***ef1α***  ENSDARG00000039502 | CCTGGGAGTGAAACAGCTG | GCCTCCAGCATGTTGTCAC |
| ***opn1sw1***  ENSDART00000045677 | GTCGCCATCCCTGCTTTCTT | CAGTCTCCATGATGCAGGCG |
| ***opn1sw2***  ENSDART00000017274 | TGACCTGAGACTGGCAACC | TGGCGCAACAGAGGAGA |
| ***opn1mw1***  ENSDART00000097008 | GGGTTCCCTATGCCAGCTT | AAGGGTGTTGAGCATGCAGC |
| ***opn1lw2***  ENSDART00000044861 | CATGCCTGCCTACTTTGCCA | GCAGGAGCCACAGAAGACAC |
| ***rho***  ENSDART00000002193 | CGGTGTCAACAACGAGTCCT | GCAACCAGCAGATGAGGAAG |
| ***ascl1a***  ENSDART00000038386 | GCCAGACGGAACGAGAGAGA | AGGGTTGCAAAGCCGTTG |
| ***lin28a***  ENSDART00000004328 | GTCTGGACTCACCCGTGGATG | CTCCAGGCCCCGTCACTTGTA |
| ***stat3***  ENSDART00000022712 | GAGGAGGCGTTTGGCAAA | TGTGTCAGGGAACTCAGTGTCTG |
|  |  |  |

**Supplementary Table 2. List of antibodies used in the immunofluorescence analysis**

| **Primary Antibody** | **Host** | **Dilution** | **Supplier** | **Reference** |
| --- | --- | --- | --- | --- |
| Anti-Yap FL (63.07) | Mouse | 1:100 | Sta Cruz Biotechnology | 101199 |
| Anti-GFP | Rabbit | 1:100 | Invitrogen | A11122 |
| Anti-GFP | Mouse | 1:100 | Roche | 11814460001 |
| Anti-Glutamine Synthetase | Mouse | 1:200 | BD Biosciences | 610517 |
| Anti-Zpr-1 | Mouse | 1:100 | ZIRC | ZIRC |
| Anti-DsRed | Rabbit | 1:100 | Clontech | 632496 |
| PCNA | Rabbit | 1:100 | Sta Cruz Biotechnology | FL-261 |
| Sox2 | Rabbit | 1:200 | GeneTex | GTX124477 |
| **Secondary Antibody** | **Host** | **Dilution** | **Supplier** | **Reference** |
| Alexa 488 | Goat (anti-rabbit) | 1:500 | Invitrogen | A11008 |
| Alexa 488 | Goat (anti-mouse) | 1:500 | Invitrogen | A11001 |
| Alexa 647 | Donkey  (anti-rabbit) | 1:250 | Jackson Immunoresearch | 711-605-152 |
| Alexa 647 | Donkey  (anti-mouse) | 1:250 | Jackson Immunoresearch | 715-605-151 |

**Supplementary Table 3. List of the number of retinas used in quantitative experiments**

| **Figure** | **Marker** | **Number of retinas used** |
| --- | --- | --- |
| **1** | *careg* | . 12hpl, controls and *careg*,DN-Yap+ n=10, each  . 1dpl, controls and *careg*,DN-Yap+ n=14, each |
| **2** | PCNA  EdU | . 1dpl, controls n=12 and DN-Yap+ n=11  . 2dpl, controls and DN-Yap+ n=20, each  . 3dpl, controls and DN-Yap+ n=21, each  . 1dpl, controls n=12 and DN-Yap+ n=13  . 2dpl, controls and DN-Yap+ n=12, each  . 3dpl, controls and DN-Yap+ n=11, each |
| **3** | Sox2 | . 6dpf (no lesion) *gfap*,DN controls n=10  . 1dpl, controls n=7 and *gfap*,DN-Yap+ n=10  . 2dpl, controls n=10 and *gfap*,DN-Yap+ n=8  . 3dpl, controls n=13 and *gfap*,DN-Yap+ n=19 |
| **S2** | *careg*  *careg*;PCNA | . 12hpl, controls and *careg*,CA-Yap+ n=12, each  . 1dpl, controls n=11 and *careg*,CA-Yap+ n=10  . 2dpl, controls n=14 and *careg*,DN-Yap+ n=13  . 2dpl, controls n=10 and *careg*,DN-Yap+ n=14 |
| **S4** | Sox2  PCNA | . 1dpl, both controls and CA-Yap+ n=15, each  . 2dpl, both controls and CA-Yap+ n=15, each  . 3dpl, both controls and CA-Yap+ n=8, each  . 2dpl, both controls and CA-Yap+ n=17, each |
