# Supplementary material for: Yap Regulates Müller Glia Reprogramming in Damaged Zebrafish Retinas
Source: Front Cell Dev Biol. 2021 Sep 20;9:667796. doi: 10.3389/fcell.2021.667796 (PMC8488126; doi:10.3389/fcell.2021.667796)
Supplement: Supplementary file 1 [file Data_Sheet_1.zip › Lourenco et al. 2021 Supplementary information.pdf]

Supplementary Material

Supplementary Figures

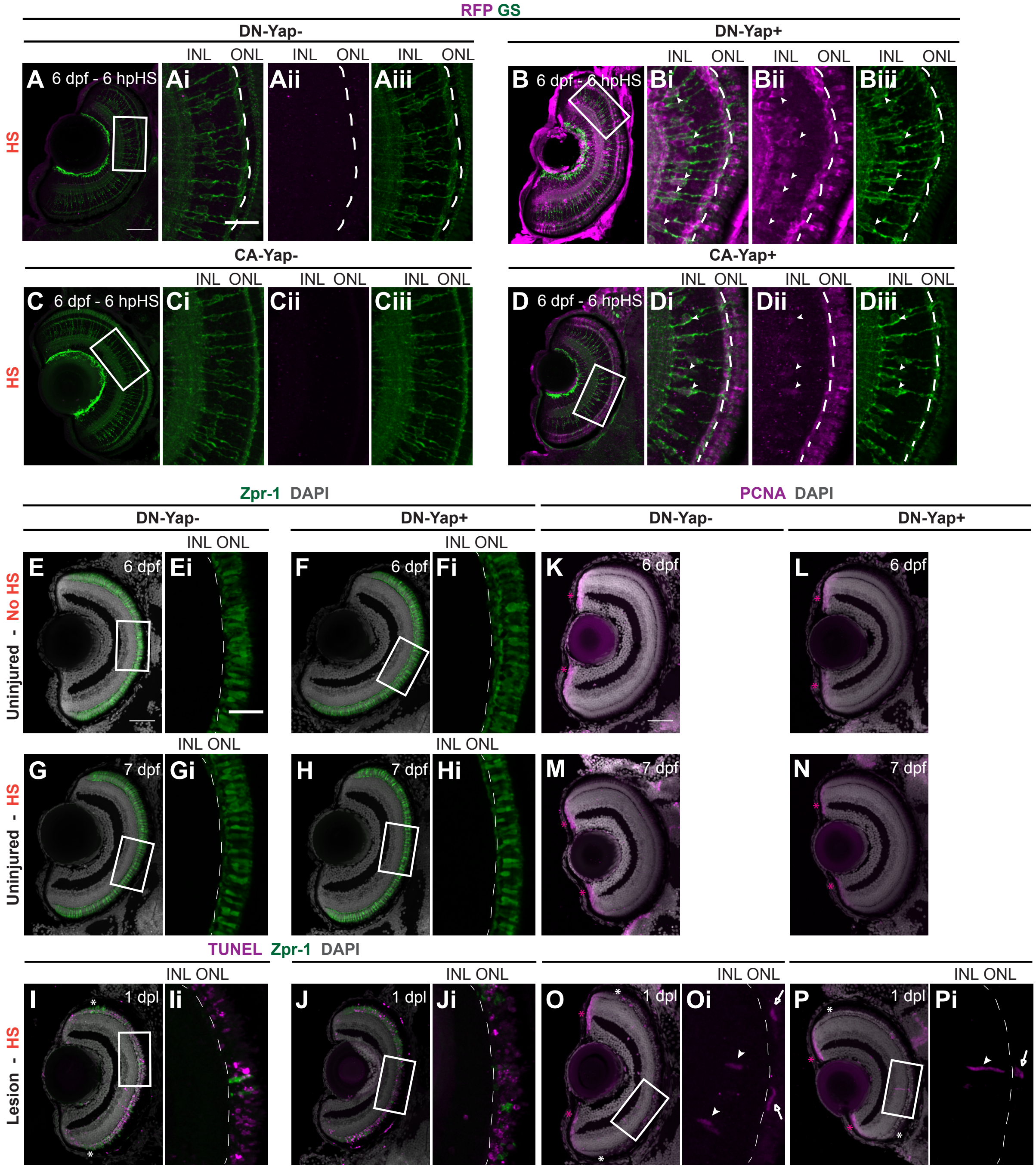

Supplementary Figure 1. Heat-shock assay does not affect differentiated photoreceptor or retina proliferation. (A-D) Transverse cryosections of 6 dpf DN-Yap – **(A,Ai-Aiii)**, DN-Yap + **(B,Bi-Biii)**, CA-Yap – **(C,Ci-Ciii)** and CA-Yap + **(D,Di-Diii)** retinas subjected to a HS for 6 h, immunostained for RFP (magenta) and glutamine synthetase (GS) (green). DN-Yap – and CA-Yap – retinas show no RFP immunostaining (magnified panels Aii,Cii, respectively), while DN-Yap + and CA-Yap + retinas present transgenes expression, RFP-positive cells, through the different layers of the retina (magnified panels Bii,Dii, respectively). This is indicative of successful DN-Yap and CA-Yap transgenes induction upon HS and expression in MGs (white arrowheads in magnified panels Bi-Biii,Di-Diii, respectively). **(E-P)** Transverse cryosections of uninjured 6 dpf DN-Yap – controls **(E,Ei,K)** and DN-Yap + (F,Fi,L) retinas not subjected to HS; and uninjured 7dpf DN-Yap – control **(G,Gi,M)** and

DN-Yap + **(H,Hi,N)** retinas subjected to a HS at 6 dpf. Retinas were immunostained for Zpr-1 (green) **(E-H)** or for proliferating cell nuclear antigen (PCNA) (magenta) **(K-N)**, and all counterstained with DAPI (grey). 1 dpl DN-Yap – control **(I,Ii,O)** and DN-Yap + **(J,Ji,P)** retinas subjected to UV light lesion and HS at 6 dpf. Retinas were labelled with TUNEL (magenta) and immunostained for Zpr-1 (green) **(I,J)** or immunostained for PCNA (magenta) **(O,P)**, and all counterstained with DAPI (grey). Pink asterisks indicate proliferating cells in the ciliary marginal zone (CMZ). White asterisks delimitate the lesioned region. White boxes delimitate magnified **(Ai-Hi,Ii-Pi)**. Scale bars correspond to 50 µm in **(A,E,K)** and 20 µm in magnified **(Ai,Ei)**.

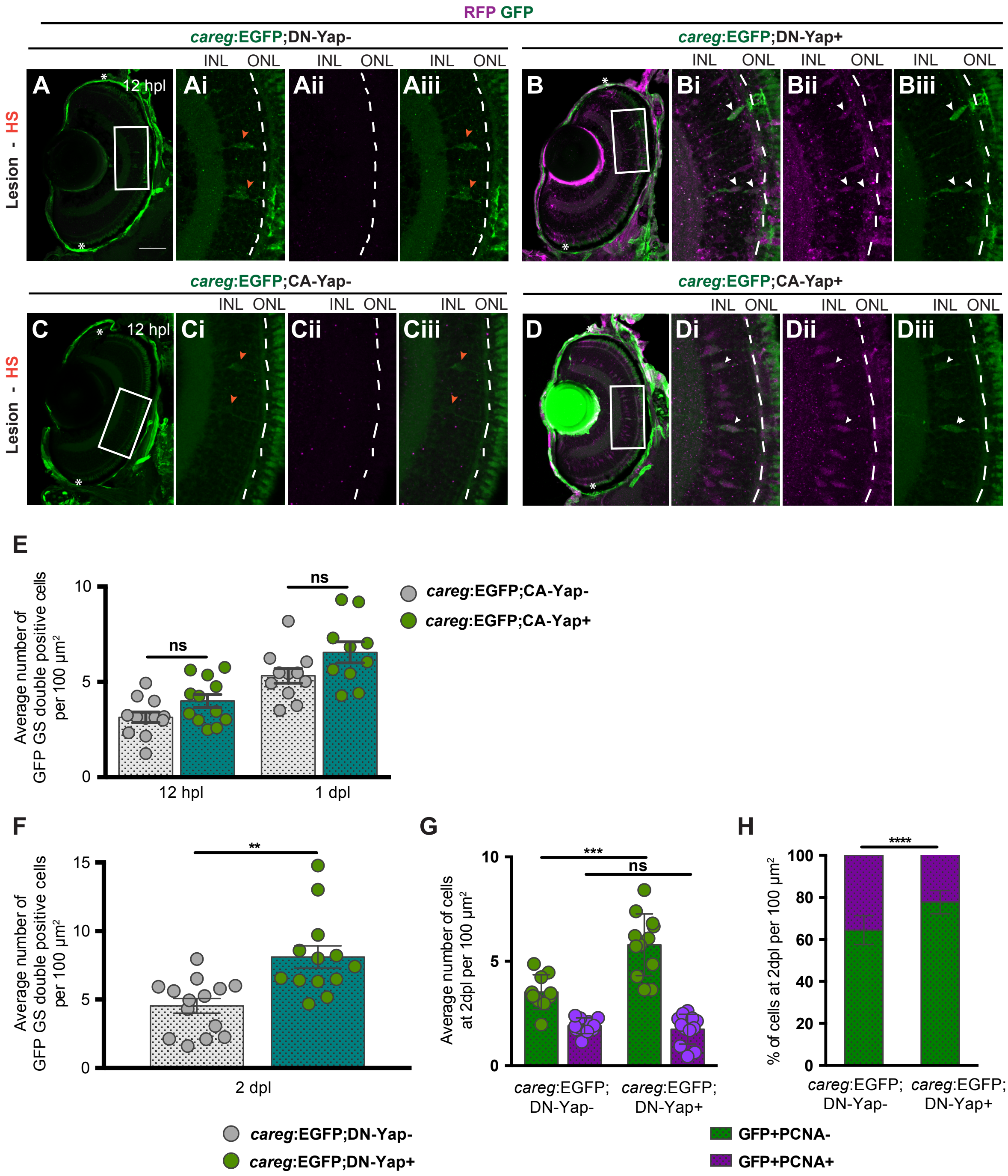

Supplementary Figure 2. DN-Yap and CA-Yap transgenes expression in activated MGs following HS. (A-D) Transverse cryosections of 12 hpl *careg*:EGFP;DN-Yap – control **(A,Ai-Aiii)**, *careg*:EGFP;DN-Yap + **(B,Bi-Biii)**, *careg*:EGFP;CA-Yap – control **(C,Ci-Ciii)** and *careg*:EGFP;DN-Yap + **(D,Di-Diii)** retinas immunostained for RFP (magenta) and green fluorescent protein (GFP) (green). DN-Yap – and CA-Yap – retinas show *careg*:EGFP+ cells (orange arrowheads in magnified Ai,Aiii,Ci,Ciii respectively) with no RFP immunostaining (magnified Aii,Cii, respectively), while DN-Yap + and CA-Yap + retinas present transgenes expression, RFP-positive cells, colocalizing with *careg*:EGFP+ cells (white arrowheads in magnified Bi-Biii,Di-Diii, respectively). This is indicative of successful DN-Yap and CA-Yap transgenes induction upon HS and specific expression in activated MGs. **(E)** Quantification of the number of double GFP GS-positive cells in *careg*:EGFP;CA-Yap – controls and *careg*:EGFP;DN-Yap + larvae retinas from 12 hpl to 1 dpl. **(F)** Quantification of the number of double GFP GS-positive cells in *careg*:EGFP;DN-Yap – controls and *careg*:EGFP;DN-

Yap + larvae retinas at 2 dpl. **(G)** Quantification of the number of GFP+PCNA- and GFP+PCNA+ cells in *careg*:EGFP;DN-Yap – controls and *careg*:EGFP;DN-Yap + larvae retinas at 2 dpl. **(H)** Percentage of GFP+PCNA- and GFP+PCNA+ cells in *careg*:EGFP;DN-Yap – controls and *careg*:EGFP;DN-Yap + larvae retinas at 2 dpl. ns, non-significant; **P < 0.01, ***P < 0.001, **** P < 0.0001; Unpaired *t*-test with Welch's correction **(E,F)**. Mann-Whitney test in **(G,H)**. White boxes delimitate magnified **(Ai-Diii)**. Scale bars correspond to 50 μm in **(A)** and 20 μm in magnified **(Ai)**. Asterisks delimitate the lesioned region.

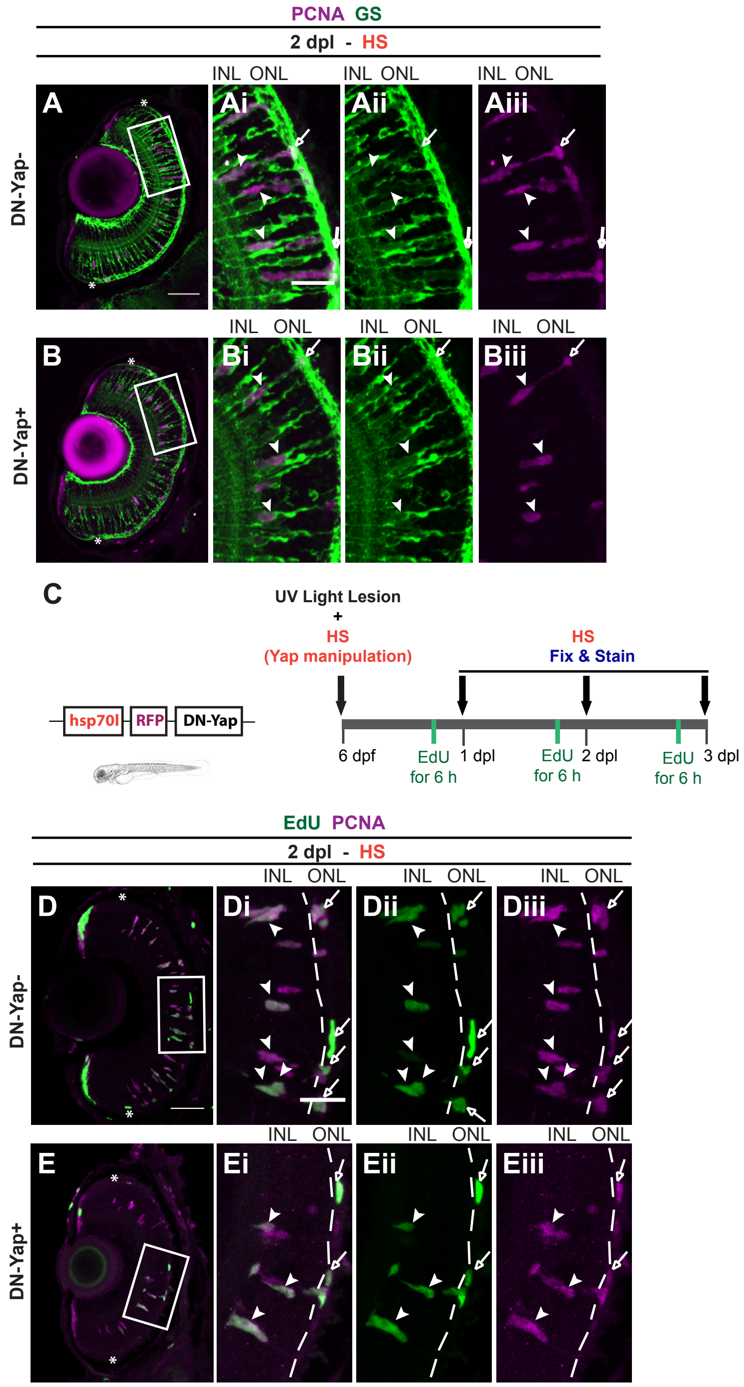

Supplementary Figure 3. MGs proliferate after photoreceptor light lesion and upon Yap inhibition. (A-B) Transverse cryosections of 2 dpl DN-Yap – control **(A,Ai-Aiii)** and DN-Yap + **(B,Bi-Biii)** retinas immunostained for proliferating cell nuclear antigen (PCNA) (magenta) and glutamine synthetase (GS) (green). Proliferating MGs are observed in the inner nuclear layer (INL) (white arrowheads) and in the outer nuclear layer (ONL) near MG endfeet (arrows) **(magnified Ai-Biii)**. **(C)** Schematic representation of EdU administration to the medium in the UV light lesion assay, 6 h before larvae fixation. **(D,E)** Transverse cryosections of 2 dpl DN-Yap – controls **(D,Di-Diii)** and DN-Yap + **(E,Ei-Eiii)** retinas immunostained for EdU (green) and PCNA (magenta). EdU and PCNA-double positive proliferating cell are observed in the INL (white arrowheads) and in the ONL (arrows) **(magnified Di-Eiii)**. Asterisks delimitate the lesioned region. Dashed lines delimitate INL from ONL. White boxes delimitate magnified **(Ai-Biii; Di-Eiii)**. Scale bars correspond to 50 µm in **(A,D)** and 20 µm in magnified **(Ai,Di)**.

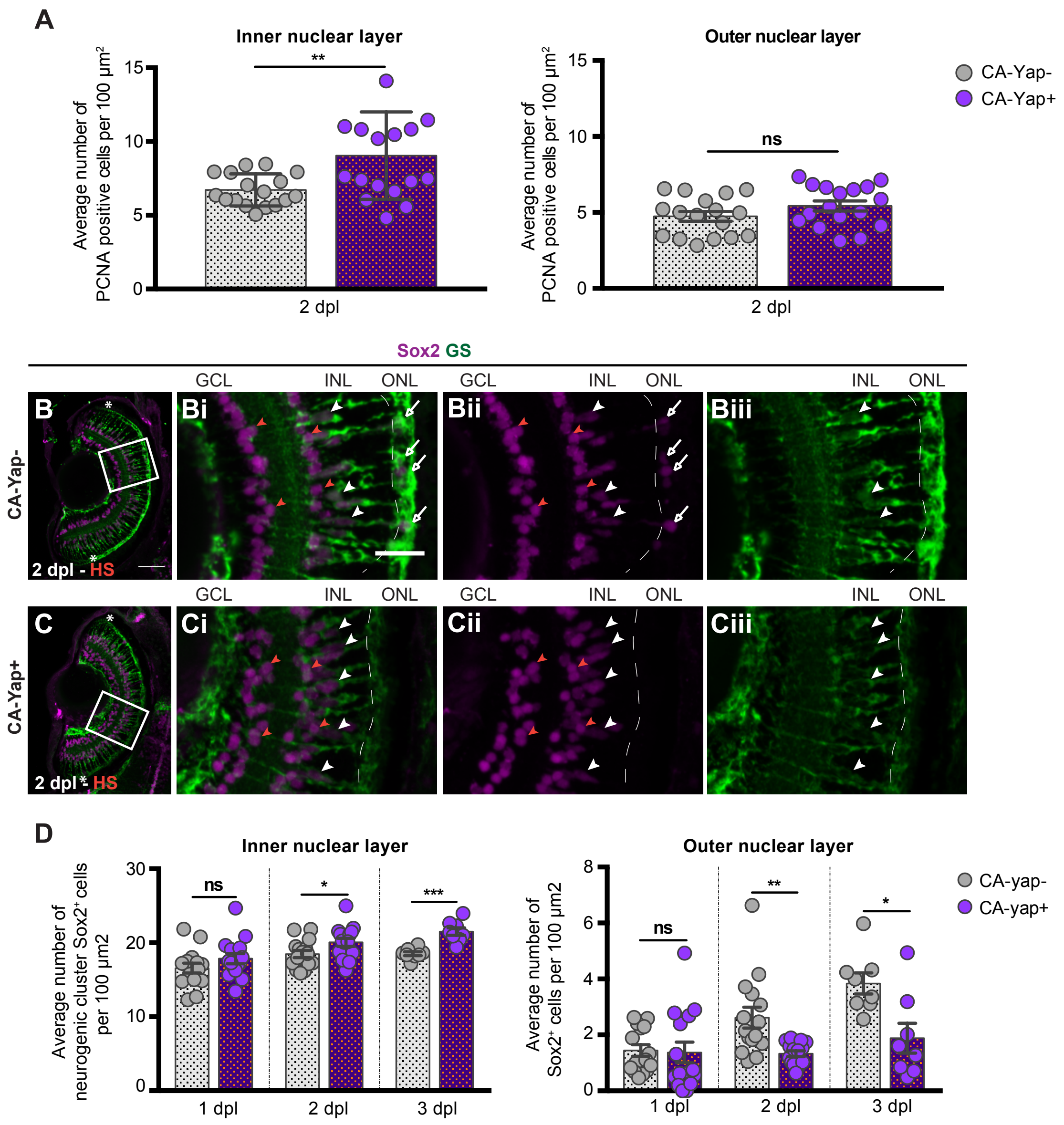

Supplementary Figure 4. Yap overexpression increases the number of proliferating MGs and Sox2-positive cells after photoreceptor light lesion. (A) Quantification of proliferating cell nuclear antigen (PCNA)-positive cells in lesioned CA-Yap – controls and CA-Yap + larvae retinas at 2 dpl, in both inner nuclear layer (INL) and outer nuclear layer (ONL). **(B-C)** Transverse cryosections of 2 dpl CA-Yap – control **(B)** and CA-Yap + **(C)** retinas subjected to UV light lesion and HS at 6 dpf, immunostained for Sox2 (magenta) and glutamine synthetase (GS) (green). Sox2 is localized in ACs, in both GCL and INL (orange arrowheads) **(magnified Bi,Bii,Ci,Cii)**, MGs in the INL (white arrowheads) and in progenitors in the ONL (white arrows) **(magnified Bi-Biii,Ci-Ciii)**. **(D)** Quantification of Sox2-positive cells in lesioned CA-Yap – controls and CA-Yap + larvae retinas from 1 to 3 dpl, in both INL and ONL. ns, non-significant; *P < 0.05, **P < 0.01, *** P < 0.001; Unpaired *t*-test with Welch's correction. White boxes delimitate magnified **(Ai-Aiii,Bi-Biii)**. Scale bars correspond to 50 µm in **(A)** and 20 µm in magnified **(Ai)**. Asterisks delimitate the lesioned region.

Supplementary Tables

Supplementary Table 1. Sequences of primers used for qRT-PCR analysis

Gene<br>Ref Ensembl (*Danio rerio*)	Forward (5'>3')	Reverse (5'>3')
ef1α<br>ENSDARG00000039502	CCTGGGAGTGAAACAGCTG	GCCTCCAGCATGTTGTCAC
opn1sw1<br>ENSDART00000045677	GTCGCCATCCCTGCTTTCTT	CAGTCTCCATGATGCAGGCG
opn1sw2<br>ENSDART00000017274	TGACCTGAGACTGGCAACC	TGGCGCAACAGAGGAGA
opn1mw1<br>ENSDART00000097008	GGGTTCCCTATGCCAGCTT	AAGGGTGTTGAGCATGCAGC
opn1lw2<br>ENSDART00000044861	CATGCCTGCCTACTTTGCCA	GCAGGAGCCACAGAAGACAC
rho<br>ENSDART00000002193	CGGTGTCAACAACGAGTCCT	GCAACCAGCAGATGAGGAAG
ascl1a<br>ENSDART00000038386	GCCAGACGGAACGAGAGAGA	AGGGTTGCAAAGCCGTTG
lin28a<br>ENSDART00000004328	GTCTGGACTCACCCGTGGATG	CTCCAGGCCCCGTCACTTGTA
stat3<br>ENSDART00000022712	GAGGAGGCGTTTGGCAAA	TGTGTCAGGGAACTCAGTGTCTG

Supplementary Table 2. List of antibodies used in the immunofluorescence analysis

Primary Antibody	Host	Dilution	Supplier	Reference
Anti-Yap FL (63.07)	Mouse	1:100	Sta Cruz Biotechnology	101199
Anti-GFP	Rabbit	1:100	Invitrogen	A11122
Anti-GFP	Mouse	1:100	Roche	11814460001
Anti-Glutamine Synthetase	Mouse	1:200	BD Biosciences	610517
Anti-Zpr-1	Mouse	1:100	ZIRC	ZIRC
Anti-DsRed	Rabbit	1:100	Clontech	632496
PCNA	Rabbit	1:100	Sta Cruz Biotechnology	FL-261
Sox2	Rabbit	1:200	GeneTex	GTX124477

Secondary Antibody	Host	Dilution	Supplier	Reference
Alexa 488	Goat (anti-rabbit)	1:500	Invitrogen	A11008
Alexa 488	Goat (anti-mouse)	1:500	Invitrogen	A11001
Alexa 647	Donkey (anti-rabbit)	1:250	Jackson Immunoresearch	711-605-152
Alexa 647	Donkey (anti-mouse)	1:250	Jackson Immunoresearch	715-605-151

Supplementary Table 3. List of the number of retinas used in quantitative experiments

Figure	Marker	Number of retinas used
**1**	*careg*	. 12hpl, controls and *careg*,DN-Yap+ n=10, each
		. 1dpl, controls and *careg*,DN-Yap+ n=14, each
**2**	PCNA	. 1dpl, controls n=12 and DN-Yap+ n=11
		. 2dpl, controls and DN-Yap+ n=20, each
		. 3dpl, controls and DN-Yap+ n=21, each
	EdU	. 1dpl, controls n=12 and DN-Yap+ n=13
		. 2dpl, controls and DN-Yap+ n=12, each
		. 3dpl, controls and DN-Yap+ n=11, each
**3**	Sox2	. 6dpf (no lesion) *gfap*,DN controls n=10
		. 1dpl, controls n=7 and *gfap*,DN-Yap+ n=10
		. 2dpl, controls n=10 and *gfap*,DN-Yap+ n=8
		. 3dpl, controls n=13 and *gfap*,DN-Yap+ n=19
**S2**	*careg*	. 12hpl, controls and *careg*,CA-Yap+ n=12, each
		. 1dpl, controls n=11 and *careg*,CA-Yap+ n=10
		. 2dpl, controls n=14 and *careg*,DN-Yap+ n=13
	careg;PCNA	. 2dpl, controls n=10 and *careg*,DN-Yap+ n=14
**S4**	Sox2	. 1dpl, both controls and CA-Yap+ n=15, each
		. 2dpl, both controls and CA-Yap+ n=15, each
		. 3dpl, both controls and CA-Yap+ n=8, each
	PCNA	. 2dpl, both controls and CA-Yap+ n=17, each
